# Novel coronavirus treatment with ribavirin: Groundwork for an evaluation concerning COVID‐19

**DOI:** 10.1002/jmv.25798

**Published:** 2020-04-10

**Authors:** Jahan S. Khalili, Hai Zhu, Nga Sze Amanda Mak, Yongqi Yan, Yi Zhu

**Affiliations:** ^1^ SystImmune Inc Redmond Washington

**Keywords:** COVID‐19, novel coronavirus, ribavirin

## Abstract

Confronting the challenge of the outbreak of COVID‐19 should sharpen our focus on global drug access as a key issue in antiviral therapy testing. The testing and adoption of effective therapies for novel coronaviruses are hampered by the challenge of conducting controlled studies during a state of emergency. The access to direct antiviral drugs, such as ribavirin, that have an existing inventory and reliable supply chain may be a priority consideration for therapies developed for the 2019‐nCoV infection outbreaks and any strain variants that may emerge. On the basis of the direct antiviral activity of ribavirin against 2019‐nCoV in vitro and evidence for potency enhancement strategies developed during the prior SARS and MERS outbreaks, ribavirin may significantly impact our ability to end the lingering outbreaks in China and slow outbreaks in other countries. The apparent COVID‐19 pandemic provides an opportunity to follow dosage guidelines for treatment with ribavirin, test new therapeutic concepts, and conduct controlled testing to apply the scientific rigor required to address the controversy around this mainstay of antiviral therapy.

## Highlights

- The multimodal anti‐viral mechanisms of action of ribavirin are described.
- Treatment of novel coronaviruses with ribavirin during SARS and MERS outbreaks are detailed.
- Challenges in evaluation of drug efficacy from viral outbreaks are described.
- Registered clinical trials to evaluate ribavirin for COVID‐19 are listed.

## 1 INTRODUCTION

The suppression of viral outbreaks is most effective when interventions are established early after the detection of a pathogen. However, novel coronaviruses (nCoV) that crossover from zoonotic hosts possess unknown sensitivities to treatments and are a principal source of pandemic risk. The clinical effectiveness of treatments from the frontlines of an outbreak can be most informative; however, the options in such an environment are limited by the shallow global pharmacopoeia of general antiviral medicines.^1^ Opportunities in this environment are further reduced by the limited inventory of antiviral medications produced by manufacturers and the accompanying supply chain optimization challenges that may delay the availability of drugs that show an early signal of efficacy. The emergence of 2019‐nCoV (officially named SARS‐CoV‐2) has demonstrated another challenge in the face of emerging nCoV outbreaks, specifically the incomplete evaluation of evidence of drug efficacy from prior nCoV outbreaks.

In this review, we critically evaluate the studies that underlie the inconclusive benefit of ribavirin for the treatment of prior nCoV outbreak strains and characterize the primary sources of the controversy. On the one hand, there persist issues of global access and medication affordability, its efficacy in general clinical practice, multimodal mechanisms of direct antiviral activity, and indirect activity of the immune system. On the other hand, there are challenges of conducting controlled clinical studies in an outbreak environment, the limitations of retrospective studies, and the absence of nCoV cases showing acute resolution of infection after treatment as well as in vitro testing data of activity against 2019‐nCoV.

## 2 ROLE FOR RIBAVIRIN IN 2019‐nCoV TREATMENT

The pathology of COVID‐19 resembles that of the 2013 MERS‐CoV and 2003 SARS‐CoV infections such that the extrapolation of treatment guidance from those prior clinical experiences can provide guidance for the current outbreak of 2019‐nCoV.^2^ The current “rapid advice guidelines for the diagnosis of and treatment of 2019‐nCoV” summarize the strong and weak recommendations for treatment on the basis of the current frontline clinical evidence from 170 confirmed cases.^3^ In this expert perspective of available data, the use of the all‐combination antiviral drug is still controversial.^3^

As strain isolates of the 2019‐nCoV are distributed for laboratory testing in cell‐based and animal model systems, recommendations for treatment may be ascribed. The first 2019‐nCoV viral strain submitted for laboratory testing was 2019BetaCoV/Wuhan/WIV04/20192 (WIV04), which was isolated from the lung fluid of one patient in a cohort of seven, six of whom worked in the proximity of the Wuhan seafood market.^4^ Indeed, the earliest report of in vitro efficacy of five FDA‐approved drugs with activity against WIV04 has been reported (ribavirin, penciclovir, nitazoxanide, nafamostat, and chloroquine). In addition, two experimental drugs (remdesivir and favipiravir) have also shown activity against WIV04.^5^ The report of in vitro direct‐acting antiviral activity against the 2019‐nCoV establishes the earliest basis for clinical guidance. Treatment with chloroquine and ribavirin may permit some advantage in an outbreak due to immediate drug availability.

Indeed, as a single agent and due to its cost and availability in China, a chloroquine phosphate multicenter trial was possible, and this drug showed signals of apparent efficacy against 2019‐nCoV.^6^ In contrast, the signals of efficacy from lopinavir/ritonavir were reported from a single case report from the index patient treated in Korea, whose viral titers diminished after treatment.^7^ Additional laboratory studies may enrich the understanding of synergistic combinations, and subsequent coordinated clinical experience will collectively inform treatment guidance during the 2019‐nCoV outbreak. Moreover, from a large number of controlled clinical trials, comparative effectiveness will be better understood, including an investigation to evaluate the merit of the addition of ribavirin to lopinavir/ritonavir treatment in outbreak regions (Table 1).

**Table 1:** Clinical settings evaluating the role of ribavirin for COVID‐19

Treatment	Description	Site	Ref.
Ribavirin + lopinavir/ritonavir + interferon‐β1b	Lopinavir/ritonavir, ribavirin, and IFN‐β combination for nCoV treatment NCT04276688	The University of Hong Kong	^8^
Ribavirin + lopinavir/ritonavir + IFN‐α1b	One arm in prospective, parallel‐design interventional trial ChiCTR2000029387	Chongqing Public Health Medical Center	^9^
	Only normal type nCoV patients are included		
Ribavirin + IFN‐α1b	One arm in prospective, parallel‐design interventional trial ChiCTR2000029387	Chongqing Public Health Medical Center	^9^
	Only normal type nCoV patients are included		
Physician's choice of recommended antiviral drugs including ribavirin	Clinical trial on the regularity of TCM syndrome and differentiation treatment of COVID‐19 (CTOROTSADTOC) NCT04306497	Not determined: Sponsor: Jiangsu Famous Medical Technology Co Ltd	^10^
Ribavirin	China 2019‐nCoV pneumonia diagnosis and Treatment Plan Edition 5‐Revised:	N/A	^11^
	500 mg IV BID or TID		
Ribavirin	China 2019‐nCoV pneumonia diagnosis and Treatment Plan Edition 5:	N/A	^12^, ^13^
	4 g PO loading dose		
	→ 1.2 g PO q8h		
Ribavirin, high dose	Antiviral treatment guidelines for MERS:	N/A	^12^
	2 g PO loading dose		
	→ 1.2 g PO q8h for 4 d		
	→ 0.6 g PO q8h for 4‐6 d		
Ribavirin, intermediate dose	Antiviral treatment guidelines for MERS:	N/A	^12^
	2 g PO loading dose		
	→ 10 mg/kg PO q8h for 10 d		

With the rapid transmission of 2019‐nCoV and our limited understanding of viral evolution during this process, the guidance on drug usage and testing must remain extensive. Forthcoming laboratory‐based test results on new strains and those obtained by using different models may change the order of sensitivity to available treatments for emergent strain variants. The spread of 2019‐nCoV globally is a factor that can also influence individual‐ and population‐level treatment outcomes with different therapies.^14^ The treatment options developed may lead to observations of efficacious combinations, as seen previously with the addition of ribavirin to combinations of direct antivirals and interferons (IFNs).^15^, ^16^ The early inclusion of ribavirin in clinical testing during the outbreak in China and the presence of usage guidelines are a good sign for the evaluability of efficacy in retrospective studies.

## 3 RIBAVIRIN: BASIS OF ANTIVIRAL ACTIVITY

Ribavirin is a guanosine analog that interferes with the replication of RNA and DNA viruses. However, the antiviral activity of ribavirin is not limited to interference with polymerases, that is, the structure of ribavirin also interferes with RNA capping that relies on natural guanosine to prevent RNA degradation. Moreover, to further promote the destabilization of viral RNA, ribavirin inhibits natural guanosine generation by directly inhibiting inosine monophosphate dehydrogenase in a pathway that is vital for the production of the guanine precursor to guanosine.^17^

Even when treatment incompletely blocks the virus from replicating, viral nucleic acid replication in the presence of ribavirin occurs with reduced fidelity, leading to the introduction of random mutations that can reduce the viability of the virus.^18^ This mechanism of action may overcome structure‐dependent modes of viral immune evasion in a patient and encourage the generation of protective immunity.

The indirect antiviral properties of ribavirin as mediated by the immune system were first observed in the treatment of patients with hepatitis whose symptoms improved without a reduction in the viral load.^19^ Further study of the immune cells in these patients found that the antiviral Th1 arm of the immune system was boosted by ribavirin, and additional studies have indicated that the enhanced polarization of the immune response may be at the expense of regulatory T cells that suppress the immune response.^20^, ^21^, ^22^ This mechanism of immune regulation is one rationale for the testing of ribavirin as an anticancer agent. Ribavirin's multimodal antiviral properties may limit viral replication, reducing the patient's viral load, subsequent pathological tissue damage, and the risk of transmission. There is no knowledge regarding the dosage required to experience each of the unique mechanisms of action of ribavirin, and it is also not known whether the relative threshold for the activity will vary among different patient populations and clinical contexts. A direct viral replicative inhibition is not the exclusive determinant of ribavirin's multimodal antiviral activity. Ribavirin's multiple mechanisms of action likely support its longevity and quality as a clinical resource.

As a very mature drug, with significant pharmacological research behind it, the pharmacokinetics and bioavailability data for ribavirin are available to inform dosing both as a single agent and as a part of combination therapies.^23^ The clinical experience with ribavirin in the pediatric setting for respiratory syncytial virus infection and in the chronic infection setting for hepatitis C offers a wealth of practitioner experience with its safety profile and efficacy.^24^, ^25^, ^26^ To achieve efficacy in these two distinct clinical settings, ribavirin is delivered either as an aerosol form or orally. However, concerning the use of CoV, all reports indicate IV or oral dosing.

The mean bioavailability of a 400‐mg dose of ribavirin is 51.8% ± 21.8% after an IV loading dose of 150 mg. Using a three‐compartment model for PK analysis, the mean gamma phase half‐life is 37.0 ± 14.2 hours. Ribavirin is rapidly absorbed and has a T‐max after the oral administration of 1 hour after the first dose, 1.7 hours after the second dose, and 3 hours after the multiple‐dose. The route of ribavirin elimination is renal.^23^, ^27^ The average peak serum level of ribavirin in human is 24 µg/mL after a 1000‐mg IV dose (Box 1).^28^

Box 1 **Proposal to decelerate global pandemics**

For people living in highly populated areas in regions affected by the 2019‐nCoV pandemic, there is the creation of a high‐risk environment due to the high density of multiple strains of viruses, including 2019‐nCoV. Environments that are outside the traditional medical setting require a new approach to treatment and prevention, and they represent a new aspect of nCoV pandemic control: treatment within large‐scale and high‐density quarantines of infected and noninfected individuals. To prevent either the emergence of new 2019‐nCoV strains or the spread of other viruses, the treatment of mild cases in these areas with antiviral therapy is a high priority for local and global health professionals.

We propose the usage of ribavirin in this environment for the following reasons:

1) Broad activity toward conventional and novel viruses of DNA and RNA types.
2) Multiple mechanisms of direct antiviral action.
3) Random mutagenesis of viruses to promote T cell response.
4) Indirect mechanism of action via Th1 polarization.
5) Tolerable and well‐characterized side effect profile.
6) Mature clinical experience and comprehensive demographic characterization.
7) Accessibility.
8) Affordability.

The cost for manufacturing ribavirin is US $0.20 to $2.10 per gram.^29^ An example of over‐the‐counter retail ribavirin cost in China is as follows: 1800 mg of ribavirin retail price is approximately US $1.00, and it is formulated in 50‐mg water‐soluble powder packets for oral administration.

## 4 RIBAVIRIN EXPERIENCE IN THE SARS‐CoV OUTBREAK

Ribavirin has a well‐established history of usage in emergency clinical management plans for nCoV, in which the greatest benefit has been reported with early administration upon presentation with pneumonia and before sepsis or organ system failure.^30^ This clinical utility has been signaled in small research studies on the treatment of coronaviruses during the SARS‐CoV outbreaks in China and North America, and MERS‐CoV outbreaks in the Middle East and Asia; however, no definitive clinical study has yet established a therapeutic benefit of ribavirin with 2019‐nCoV.

The global clinical experience with ribavirin delivery for the treatment of nCoV started with SARS‐CoV, for which ribavirin was initially indicated on the basis of the pathological similarity of SARS‐CoV to the acute respiratory syndrome, which requires a typical administration of ribavirin and corticosteroid.^31^, ^32^ In Hong Kong in 2003, for a reported cohort of 75 patients, the indication for the usage of antiviral therapy was after the exclusion of antibiotic therapy as a part of establishing the diagnosis of SARS. Ribavirin was administered intravenously at 8 mg/kg every 8 hours for 14 days. This treatment was combined in a regimen with intravenous hydrocortisone, then oral prednisolone, and pulses of intravenous methylprednisolone if the condition of patients worsened.^31^ In a second report from the Hong Kong outbreak, a series of 138 SARS patients were treated with ribavirin secondary to oseltamivir. The ribavirin was delivered orally at 1.2 g, three times per day in combination with prednisolone. Patients with the worsening disease received intravenous ribavirin at a dose of 400 mg every 8 hours in addition to pulsed methylprednisolone.^32^ The doses of ribavirin used in these reports were associated with a V‐shaped curve of viral load, which seemed to exclude the absence of antiviral activity.

Subsequently, in the Canadian SARS‐CoV outbreak, ribavirin was administered early with corticosteroids, and no conclusive results of efficacy could be established, despite viral and symptom flare‐up in a portion of patients after treatment cessation.^33^, ^34^ The usage of ribavirin in Canada in 2003 was based on the recommended ribavirin tapering treatment for viral hemorrhagic fever, with a loading dose of 2 g, followed by 1 g every 6 hours for the subsequent 4 days and 500 mg every 8 hours for the subsequent 4 to 6 days.^27^ This dose was significantly greater than that used in Hong Kong for SARS. In a multicenter study in the Toronto area, a series of 144 SARS patients were analyzed, of whom 126 had received this ribavirin dosing regimen schedule and 40% received additional corticosteroids.^34^

Although the reports of the lower dose ribavirin treatment schedule used in Hong Kong in 2003 did not include descriptions of adverse events, the Canadian experience with higher dosing that year provided a greater insight into the adverse effects of both ribavirin and corticosteroids. Ribavirin usage was associated with hemolysis in 76% of patients, defined as a 1.5‐fold increase in bilirubin or a decrease in haptoglobin. In 49% of patients, a 2 g/dL decrease of hemoglobin was observed. In addition, some indication of liver toxicity was indicated on the basis of elevated transaminases, defined as a 1.5‐fold increase in aspartate aminotransferase or alanine aminotransferase in 40% of patients. Acute toxicity led to the discontinuation of ribavirin in 18% of patients.^34^ The prescribing information for ribavirin indicates the expected teratogenic and carcinogenic effects of this drug class on the basis of preclinical animal model testing. The recommendation after the usage of ribavirin in the Canadian SARS outbreak was for contraceptive usage, which was to be advised for 6 months after treatment, equivalent to 15 half‐lives of nucleotide accumulation.^35^

To further complicate the evaluation of high‐dose ribavirin monotherapy is the possibility that corticosteroids may have delayed viral clearance, prolonging infections while reducing the symptomatic inflammatory cytokines.^36^, ^37^, ^38^, ^39^ Currently, these observations support the contraindication for the usage of corticosteroids for 2019‐nCoV.^12^ Moreover, the first report of outcomes from corticosteroid usage in COVID‐19 patients shows no benefit.^40^ The retrospective case reviews from the 2003 SARS‐CoV outbreaks have not allowed a robust evaluation of the therapeutic benefit of ribavirin due to the potentially deleterious effects of corticosteroids.

Upon recognition of the potential efficacy of lopinavir/ritonavir against SARS‐nCoV in vitro in 2003, the protease inhibitor lopinavir/ritonavir was combined with ribavirin. A study of 41 SARS‐CoV patients showed a favorable clinical response with lopinavir/ritonavir and ribavirin when compared with historical outcomes with ribavirin and corticosteroids.^41^ That study used the ribavirin dosing schedule for SARS in Hong Kong, which was not associated with treatment discontinuing toxicity. However, the study design included ribavirin in both the treatment and control groups, limiting interpretation of the effect of the nucleoside analog ribavirin.

Challenges in the evaluation of ribavirin activity in patients during the previous SARS‐nCoV and MERS‐nCoV outbreaks continue to leave family doctors who reside in areas of outbreak without clear answers regarding the benefit of ribavirin. Although the drug has significant activity against coronaviruses in laboratory testing, the dose required to achieve that activity in patients may not have been known in prior practices without limiting toxicities.^5^, ^41^

The coronavirus encodes RNA replication proofreading machinery that can partially resist one mechanism of action of nucleoside analogs, placing additional importance on our ability to determine therapeutic doses of ribavirin.^42^ However, this resistance does not preclude the testing of other nucleoside analogs, such as remdesivir, in cases of 2019‐nCoV.^43^ The knowledge of this mode of nCoV resistance to nucleoside analogs may merit the consideration of testing ribavirin with remdesivir to reduce the emergence of treatment‐resistant strains on the basis of mutations in the genes that encode the RNA replication machinery.^44^

## 5 RIBAVIRIN EXPERIENCE IN THE MERS‐CoV OUTBREAK

In the pursuit of better treatment of MERS‐CoV, multiple assay cell lines were used to test for antiviral activity against the strain hCoV‐EMC/2012, yielding insights into ribavirin.^28^ The IC‐50 dose of ribavirin required to achieve direct antiviral activity toward hCoV‐EMC/2012 exceeded the level achievable in humans using the standard assay cell line Vero‐RML6, for which direct antiviral activity of ribavirin is now available for 2019‐nCoV.^4^, ^45^ In this study, the LLC‐MK2 cell‐based assay was identified as a model host for the evaluation of ribavirin's antiviral properties against hCoV‐EMC/2012.^45^ Comparatively, the standard Vero‐RML6 cell‐based assay is defective in facilitating the multimodal activity of ribavirin because it is limited in its capacity to convert ribavirin into its mono‐ and triphosphate forms. The difference in the potency of single‐agent ribavirin between this Vero‐RML6 and LLC‐MK2 cell‐based assays was the difference between unachievable and achievable dose parity in human serum (EC‐50 41.45 µg/mL, EC‐90 92.15 µg/mL vs EC‐50 16.33 µg/mL, EC‐90 21.15 µg/mL, respectively). In accordance with the clinical reports from the MERS outbreak, the LLC‐MK2 cell‐based model showed that the addition of ribavirin to IFN‐α2b improved the antiviral effect by 2.16 log against hCoV‐EMC/2012.^28^ With the evaluation of additionally characterized data from in vitro models, insights from multimodal antiviral agents against 2019‐nCoV will be informative.

During the outbreak of MERS‐CoV, ribavirin was paired with either IFN‐α2b or IFN‐α2a to engage two independent mechanisms of antiviral activity. This combination was synergistic in laboratory tests, reducing the therapeutic threshold for ribavirin to block viral replication.^28^ In Saudi Arabia, an interventional study of patients presenting with MERS‐CoV, who received oral ribavirin and weekly s.c. 180 µg IFN‐α2a for 2 weeks (n = 20) vs supportive care alone (n = 24), indicated a superior survival and reduced intensive care unit admission rate in the treatment group.^16^ In that study, the dosage of oral ribavirin was maintained for 8 to 10 days, with adjustments to dosage determined on the basis of creatinine clearance. Three dose groups were administered on the basis of creatinine clearance, specified as group 1: >0.833 mL/sec/m^2^, group 2: 0.333‐0.833 mL/sec/m^2^, and group 3: <0.333 mL/sec/m^2^ or on dialysis. After receiving an initial 2000‐mg loading dose, the 10‐day ribavirin schedules for each group were as follows: group 1: 1200 mg every 8 hours for 4 days and then 600 mg every 8 hours for 4 to 6 days; group 2: 600 mg every 8 hours for 4 days and then 200 mg every 6 hours for 4 to 6 days; group 3: 200 mg every 6 hours for 4 days and then 200 mg every 12 hours for 4 to 6 days.^16^ Using this dosing schedule and in combination with weekly IFN, the ribavirin was well tolerated. Significant adverse events in the treatment group included anemia, which was determined as a twofold mean decrease in hemoglobin (4.32 vs 2.14 g/L). The discontinuation of therapy was not required.

This treatment combination for MERS‐CoV was deployed for the limited number of cases in Korea.^46^ However, a retrospective study from the primarily affected region of MERS‐CoV reviewed cases treated with ribavirin paired with IFN‐α2b, and it was unable to establish a definitive therapeutic benefit, a conclusion that was attributed to the nature of the retrospective and uncontrolled study design.^30^ To date, the 44‐patient, single‐institution experience demonstrating the benefit of ribavirin is considered to be the best evidence of a ribavirin treatment combination for coronavirus infection. In a systematic review of treatment options for MERS, the IFN‐β/ribavirin combination therapy was suggested on the basis of a positive risk‐benefit profile, whereas ribavirin monotherapy‐associated toxicity was noted and thus assumed to not likely provide sufficient benefit to outweigh the toxicity.^47^ However, the most encouraging evidence for the progressive evolution of treatment is the demonstration of tolerability of lopinavir/ritonavir, ribavirin, and IFN‐α2a in a case study of MERS‐CoV, suggesting that this combination should be tested as a treatment for 2019‐nCoV.^48^, ^49^

## 6 RIBAVIRIN EXPERIENCE IN THE 2019‐nCoV OUTBREAK

The government initially recommended the use of ribavirin in 2019‐nCoV pneumonia diagnosis in cases of China, based on Treatment Plan Edition 5, such that upon the diagnosis of pneumonia, a 4‐g oral loading dose should be delivered, followed by a 1.2‐g oral dose every 8 hours.^13^ This guidance was then modified to 500 mg IV BID or TID in the revised edition 5.^11^ Although this information may be updated as new evidence becomes available for guidance, previous experience in MERS can assist in understanding the basis of enhancing ribavirin potency toward nCoV as well as extending potential benefits by prescribing low‐ and high‐treatment options. In the first three published case series of 2019‐nCoV treatment (total of 180 cases Wuhan, China; 1 case WA), no patients have reportedly been treated with ribavirin.^12^, ^50^, ^51^ However, the announcement of new clinical studies will offer new evidence of the role of ribavirin in clinical practice for 2019‐nCoV^8^, ^9^, ^10^, ^11^, ^12^ (Table 1).

Although significant effort and resources are contributed to the research and development of nCoV treatments, in times of outbreak, care and preparation are required to apply a scientific approach to quantify the therapeutic benefit of medicines that are already available during such viral outbreaks. On the basis of these prior clinical experiences and others, controlled studies are underway to evaluate the available courses of therapy for COVID‐19. Among the possible studies that should be completed are drug combinations that use the widely available ribavirin.^52^ Encouraging signals for the well‐documented antiviral ribavirin are the demonstration of in vitro antiviral activity toward the WIV04 strain of 2019‐nCoV, its well‐established management of side effects, and the potential for lower dosing, based on treatment synergies.^5^ In the face of this public health emergency, we are mindful of the risk of a deluge of clinical trials that may impact the recruitment and evaluability of prospective research.

The public good requires that patients can access life‐saving treatments for infectious diseases in an affordable and timely fashion. Access to medical products and establishing their therapeutic benefit are both essential to meet this obligation. Effective clinical treatments prepared from the existing pharmacopoeia can save many lives and achieve the greatest benefit for the public while facing the challenge of 2019‐nCoV and future nCoV strains.

## 7 SUMMARY AND CONCLUSION

The wide availability and low cost of ribavirin support its potential to significantly impact the treatment of nCoV infections. The challenges in the evaluation of ribavirin efficacy from 2003 during SARS and the 2013 MERS outbreaks led to a summary evaluation of its utility as controversial in the treatment of COVID‐19 patients. A large number of clinical studies and retrospective analyses that will come from the 2019‐nCoV outbreak will put the controversy of ribavirin efficacy in a broader context. For ribavirin and myriad other treatments, both the clinical results and quality of evidence will reveal the challenges that face frontline physicians who treat patients in a medical setting and evaluate prophylaxis for novel high‐risk environments formed by large quarantined populations. The critical need for treatment and patient care in outbreak settings, on the frontlines of nCoV outbreaks, will place stress on any medical system and clinical research mechanism. However, controlled clinical studies are underway to permit a prospective evaluation of efficacy, and the government Treatment Plan Edition 5 and revised and prescribed usage guidelines distributed in China will assist in the comparability of multicenter experiences in retrospective analyses. The efforts of clinical research professionals will help both in this outbreak and future outbreaks of nCoV, which will possess unknown sensitivities to our antiviral pharmacopoeia.

## CONFLICTS OF INTEREST

The authors disclose that their affiliated organizations will support clinical trials with ribavirin for the treatment of novel coronavirus infections.
